# Long-term preservation of muscle function and structure by repeated administration of cardiosphere-derived cells in *mdx* mice

**DOI:** 10.1016/j.stemcr.2025.102468

**Published:** 2025-03-20

**Authors:** Russell G. Rogers, Jack Antich, Mario Fournier, Ariel Omidfar, Lizbeth Sanchez, Juliet Alfaro, Jonah Zarrow, Nancy Manriquez, Alessandra Ciullo, Jackelyn Valle, Eduardo Marbán

**Affiliations:** 1 Smidt Heart Institute, Cedars-Sinai Medical Center, Los Angeles, CA 90048, USA

**Keywords:** cardiosphere-derived cells, cell therapy, Duchenne muscular dystrophy, extracellular vesicles, regenerative medicine

## Abstract

Duchenne muscular dystrophy (DMD) is a progressive myodegenerative disease that leads to severe muscle weakness and premature death. Mouse cardiosphere-derived cells (mCDCs) and extracellular vesicles (EVs) secreted by human cardiosphere-deriveds (hCDC-EVs) are therapeutic to mice with advanced-stage DMD. Here, we investigated the long-term benefits of monthly dosing when initiated early. At the endpoint, exercise performance and skeletal muscle function were strikingly preserved in *mdx* mice that had received mCDCs, but not in vehicle control. In contrast, the beneficial effects of hCDC-EVs waned after 6 months, in parallel with the development of anti-hCDC-EV antibodies. Further investigation showed that mCDCs lowered fibrosis and initiated a myogenic response program in *mdx* skeletal muscle. Thus, early and sustained intervention with mCDCs prevents disease progression for up to 1 year in *mdx* mice. This discovery offers new insights into how cell therapy can be used to treat DMD and motivates clinical testing of CDCs beginning in newly diagnosed DMD.

## Highlights

• Exercise performance and muscle function decline in *mdx* mice during aging
• Repeated dosing of mCDCs protects against functional decline in aging *mdx* mice
• mCDCs attenuate muscle fibrosis and initiate a regenerative gene program
• Repeated dosing of hCDC-EVs triggers an immunological response in *mdx* mice

## Teaser

Duchenne muscular dystrophy (DMD) is a progressive disease associated with functional decline. Chronic administration of cardiosphere-derived cell therapy prevents disease-related functional decline for up to 1 year in mice with DMD. Our findings offer new insights into the treatment of DMD.

## Introduction

Duchenne muscular dystrophy (DMD) is a progressive X-linked degenerative disease of striated musculature. DMD stems from mutations in the gene encoding dystrophin, resulting in deficiency of this “shock absorber” protein that stabilizes the cell membrane during contraction (Duan et al., 2021). The resulting chronic muscle damage is manifested as inflammation, degeneration, and fibrosis (Duan et al., 2021). Patients with DMD experience early onset skeletal muscle weakness, loss of ambulation, and cardiorespiratory failure (Sussman, 2002). We have previously shown that mouse cardiosphere-derived cells (mCDCs), a type of cardiac progenitor/stromal cell, or secreted extracellular vesicles (EVs) from human cardiosphere-derived cells (hCDC-EVs) exert robust disease-modifying bioactivity in aged *mdx* mice, when delivered intravenously (Rogers et al., 2019). The former finding motivated two clinical trials, HOPE-2 (McDonald et al., 2022) and the ongoing HOPE-3 (NCT05126758, clinicaltrials.org), testing safety and efficacy of allogeneic hCDCs (now known as deramiocel) in advanced-stage DMD. Here, we questioned whether mCDCs or hCDC-EVs might halt disease progression if initiated early and administered monthly for 1 year; thus, we tested the long-term effects of early, repeated infusion of mCDCs or hCDC-EVs to *mdx* mice. mCDCs markedly preserved muscle function and structure for up to 1 year. In contrast, hCDC-EVs became less effective after 6 months of intervention, coinciding with the development of anti-hCDC-EV immunoglobin G (IgG) antibodies.

## Results

### Repeated mCDC therapy preserves exercise capacity and muscle function up to 1 year

**Figure 1:**
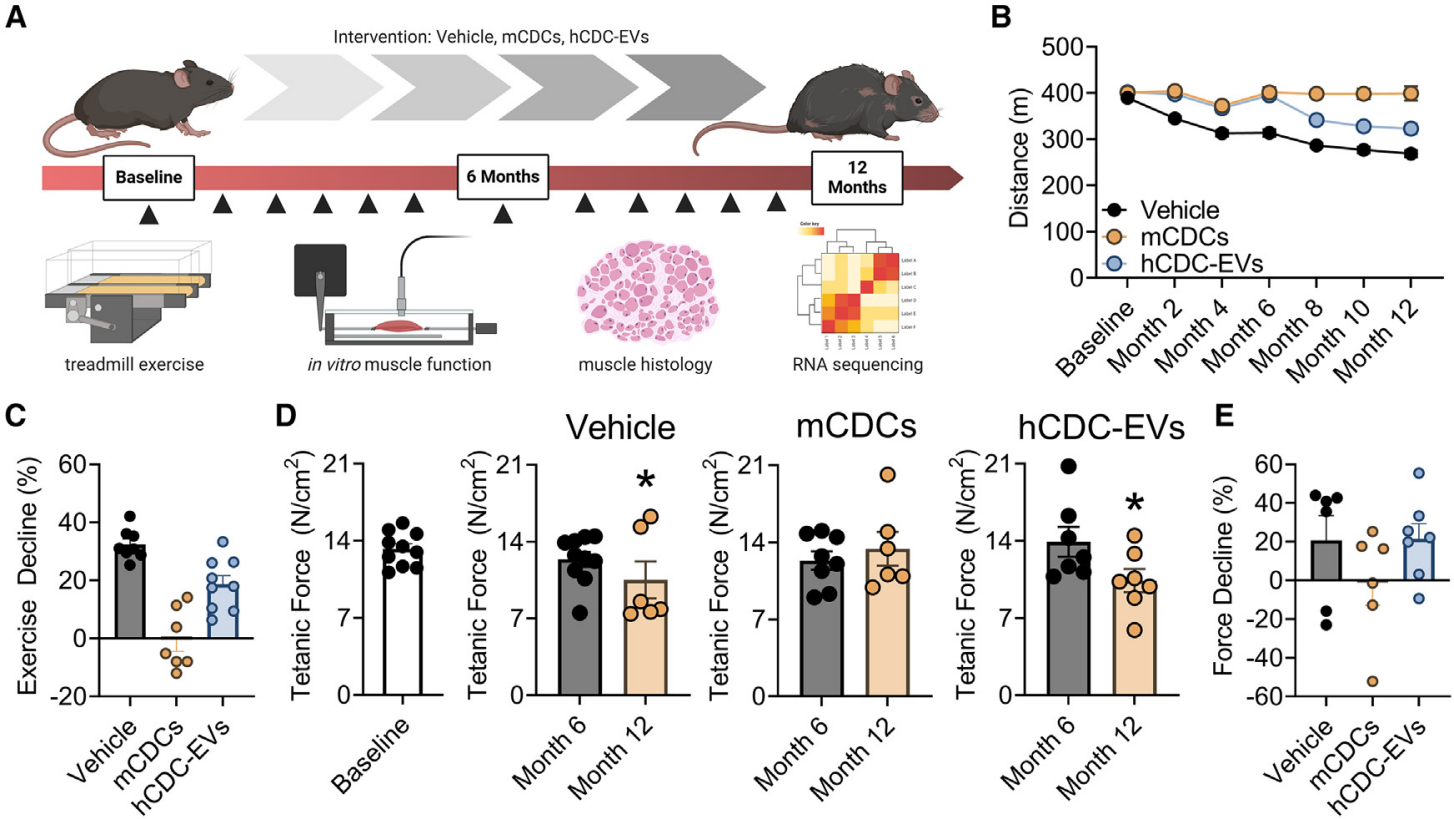
Protection against functional decline in *mdx* mice by repeated CDC therapy (A) Schematic of the study design repeatedly delivering vehicle, mCDCs, or hCDC-EVs. Schematic was made using BioRender.com. (B and C) Graded exercise test in *mdx* mice that received vehicle, mCDCs, or hCDC-EVs. Bi-monthly recording of maximally accumulated distance (B) and functional decline as a percentage of baseline (C). (D and E) *In vitro* skeletal muscle function in *mdx* mice that had received vehicle, mCDCs, or hCDC-EVs. Baseline, mid- and end-point measurements of tetanic force (D) and functional decline as a percentage of baseline (E). Physiological experiments were performed on consecutive days with *n* = 6–10 per condition or time point. Data are represented as mean ± standard error of the mean. ∗, *p* ≤ 0.05.

Cardiosphere-derived cells (CDCs) work by secreting EVs, which target inflammation, muscle degeneration, and fibrosis (Aminzadeh et al., 2018), key pathological processes active in mice and humans with DMD. A single intravenous infusion of mCDCs, or hCDC-EVs, to aged *mdx* mice sufficed to induce functional and structural benefits (Rogers et al., 2019), but the effects of repeat dosing to young *mdx* mice were unknown. Here, we examined the benefits of monthly intravenous infusions of mCDCs or hCDC-EVs in *mdx* mice starting at 8 weeks of age, after random assignment to 1 of 3 experimental groups (vehicle [control], mCDCs, or hCDC-EVs; Figure 1A). Prior to intervention, baseline measurements of maximal exercise capacity showed no differences in maximal exercise capacity among groups (Figure 1B). During the 12-month study period, maximal exercise capacity declined by 32% in the vehicle group, reflecting the natural history of skeletal myopathy in *mdx* mice (Figures 1B and 1C). In contrast, *mdx* mice that had received mCDCs showed a robust preservation of exercise capacity throughout the 12-month study period (Figures 1B and 1C). Likewise, hCDC-EVs preserved exercise capacity for the first 6 months of intervention, but the benefits faded entirely during the second half of the study (Figures 1B and 1C). To determine if repeated administration of mCDCs or hCDC-EVs to young *mdx* mice also benefited skeletal muscle, we examined the maximal force-producing capacity of the soleus—a hindlimb skeletal muscle involved in rodent locomotion. Relative to baseline, *in vitro* isometric tetanic force declined by 20% in solei from control *mdx* mice (Figures 1D and 1E), a decline in muscle function that was countered by mCDCs (but not hCDC-EVs) at the study endpoint (Figures 1D and 1E). Together, these data demonstrate profound protection against progressive functional deterioration in *mdx* mice by repeated intravenous administration of mCDCs.

### mCDCs suppress muscle fibrosis and activate a regenerative gene program

**Figure 2:**
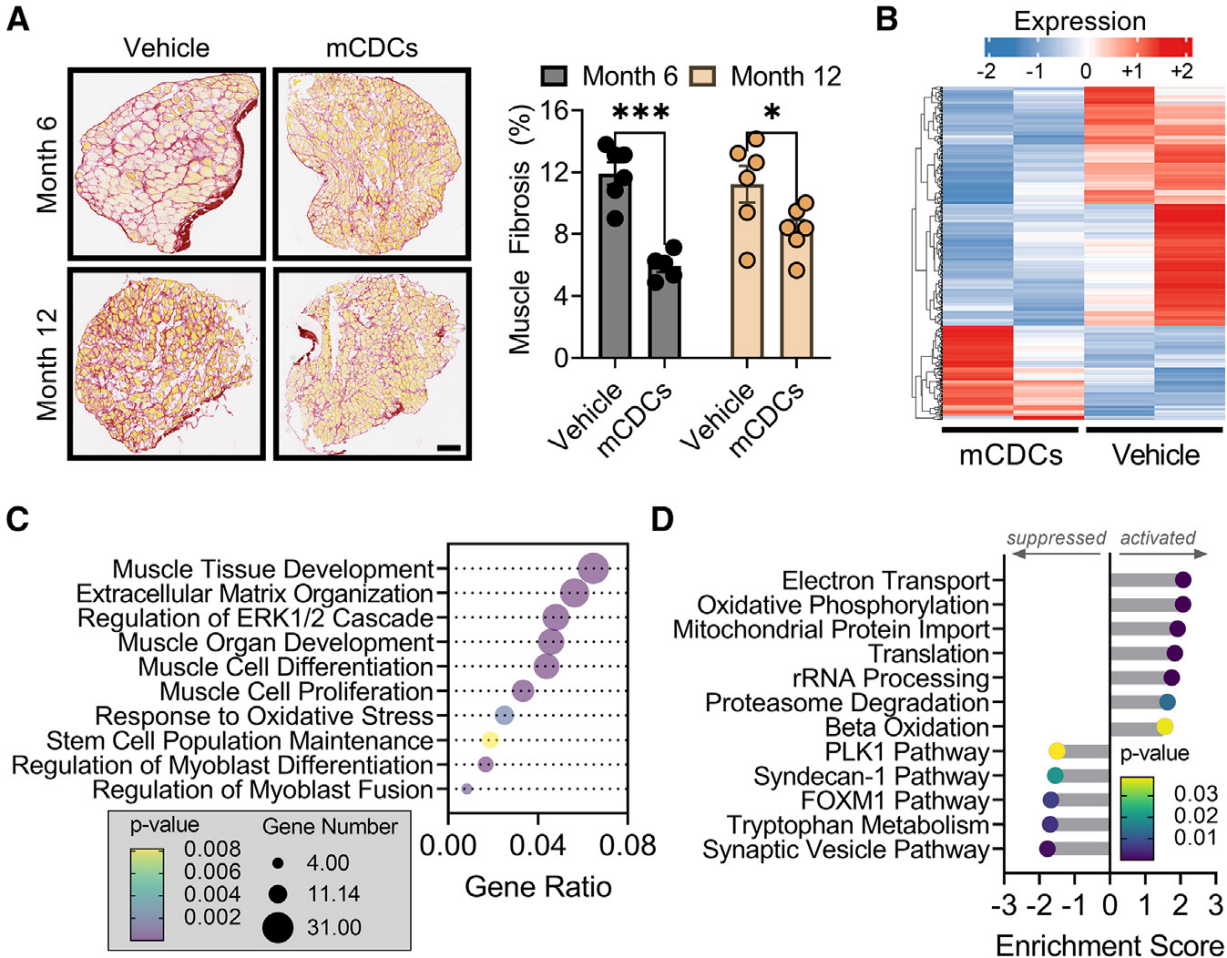
Histological and gene expression changes induced by repeated CDC therapy (A) Picrosirius red staining of skeletal muscle from *mdx* mice that had received vehicle or mCDCs at the study mid- and end-point. Scale bar, 200 μm. (B) Heatmap of bulk RNA-seq on skeletal muscle from *mdx* mice that had received vehicle or mCDCs at the study endpoint. (C) Gene Ontology analysis of bulk RNA-seq data (B). (D) Gene Set Enrichment Analysis of bulk RNA-seq data (B). Histological experiments were performed on all specimens at once with *n* = 5–6 per condition or time point. RNA-seq was performed once with *n* = 2 per condition. Data are represented as mean ± standard error of the mean. ∗, *p* ≤ 0.05; ∗∗∗, *p* ≤ 0.001.

Skeletal muscle fibrosis impairs muscle function and regeneration and is a major hallmark of muscular dystrophies (Mahdy, 2019). At 6 months, fibrotic scar accounted for 11% of the total muscle area, which persisted until the 12-month endpoint in control muscles (Figure 2A). In contrast, skeletal muscle from *mdx* mice that had received mCDCs showed a marked reduction in fibrotic scar at the 6- and 12-month endpoints (Figure 2A). These data reveal that mCDCs may antagonize pathways actively contributing to the development of fibrosis. To determine what pathways are affected, we performed bulk RNA sequencing (RNA-seq) on skeletal muscle from *mdx* mice that had received mCDCs or vehicle for 12 months. Relative to vehicle, 152 genes were up-regulated, and 384 genes were down-regulated, in muscles from *mdx* mice that had received mCDCs (Figure 2B). Many of the up-regulated genes are involved in the myogenic response to injury. Gene Ontology analysis of RNA-seq data revealed high enrichment scores for “muscle cell proliferation,” “muscle cell differentiation,” “stem cell population maintenance,” and “regulation of myoblast fusion” (Figure 2C). Gene Set Enrichment Analysis indicated that mCDCs regulate cellular processes involved in muscle repair and showed activation of metabolic pathways, including beta oxidation, electron transport, and oxidative phosphorylation (Figure 2D). Collectively, these data reveal the effects of CDCs on endogenous skeletal muscle repair mechanisms and metabolism, which may underlie therapeutic efficacy.

### IgG-mediated antibody response to hCDC-EVs

**Figure 3:**
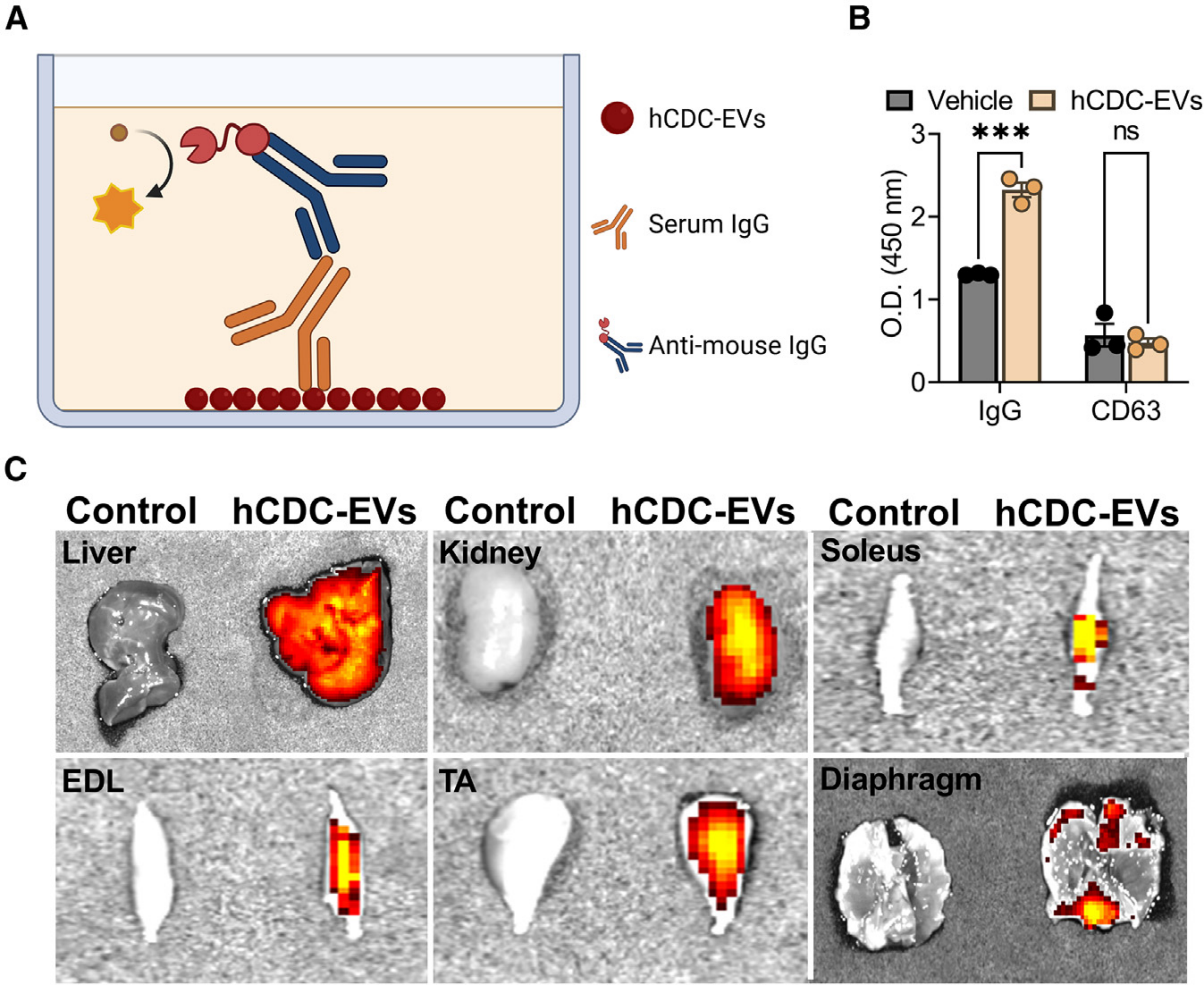
Immunoglobin G reactivity to repeated administration of human CDC-EVs (A) Schematic of the experimental design testing immunoglobin G reactivity to hCDC-EVs. Schematic was made using BioRender.com. (B) Optical density at 450 nm recorded by a microplate reader. (C) *Ex vivo* optical imaging of hCDC-EVs 24 h following intravenous injection into *mdx* mice. EDL, extensor digitorum longus; TA, tibialis anterior. Enzyme-linked immunosorbent assay was performed once with *n* = 3 per condition. Data are represented as mean ± standard error of the mean. ∗∗∗, *p* ≤ 0.001; ns, not significant.

EVs have been generally regarded as non-immunogenic (Saleh et al., 2019), and hCDC-EVs are therapeutic in mice (Aminzadeh et al., 2018; Rogers et al., 2019), rats (De Couto et al., 2017), and pigs (Dawkins et al., 2022). However, none of these studies explored the effects of long-term repeated dosing of hCDC-EVs. Because of the divergence in therapeutic benefits between mCDCs and hCDC-EVs after 6 repeated infusions (Figure 1), we questioned whether *mdx* mice had developed an immunological response to hCDC-EVs, possibly rationalizing the diminished efficacy over time. To probe such an effect, we designed an indirect enzyme-linked immunosorbent assay to measure mouse IgG immunoreactivity to hCDC-EVs (see Figure 3A for experimental design). No differences were detected in the level of CD63 (an EV marker) between groups (Figure 3B), indicating similar levels of EV coating. Relative to sera from vehicle control *mdx* mice, sera from *mdx* mice that had received hCDC-EVs contained mouse IgG antibodies that reacted with hCDC-EVs (Figure 3B). These data demonstrate immunization to xenogeneic EVs, which likely resulted in clearance by the immune system and the eventual decline in therapeutic benefits.

### hCDC-EVs home to dystrophic mouse skeletal muscle

Biodistribution studies show that EVs, when administered intravenously to animals, primarily accumulate in the liver, spleen, lungs, and kidneys within the first hour and are maintained for at least 24 h (Kang et al., 2021). In contrast, few EVs are detected in skeletal muscle at any time point within the first 24 h in otherwise healthy animals (Kang et al., 2021). To be effective, EVs need to home to dystrophic skeletal muscle where bioactivity presumably occurs. Therefore, we probed biodistribution of fluorescently labeled hCDC-EVs administered intravenously to *mdx* mice. Twenty-four hours after administration, tissues were collected, and fluorescence was detected *ex vivo* by optical imaging. Relative to unlabeled hCDC-EV controls, fluorescently labeled hCDC-EVs were detected in liver, kidneys, and various skeletal muscles—with preference given to the soleus, extensor digitorum longus, and tibialis anterior (Figure 3C). Although hCDC-EVs home to dystrophic skeletal muscle in *mdx* mice that lack anti-hCDC-EV antibodies, it is currently unclear how the development of such antibodies (Figure 3B) affects their biodistribution.

## Discussion

Although the root cause of DMD is an underlying genetic defect, processes secondary to dystrophin deficiency (e.g., inflammation, degeneration, and fibrosis) lead to worsening outcomes as the disease progresses. CDCs work by attacking disease-promoting processes to slow or even reverse the progression of DMD. We have shown that single infusions of mCDCs or hCDC-EVs suffice to improve hallmark features of dystrophic muscle in aged *mdx* mice (Aminzadeh et al., 2018; Rogers et al., 2019), an effect that can be sustained after a second injection (Aminzadeh et al., 2018). These discoveries formed the preclinical basis of two completed (McDonald et al., 2022; Taylor et al., 2019) and one ongoing (HOPE-3) clinical trials. While the prior work showed the benefits of 1 or 2 doses of mCDCs or hCDC-EVs in mice with advanced-stage DMD, the effects of repeated dosing prior to major functional deterioration remained unknown. Here, we explored the effects of long-term administration of mCDCs or hCDC-EVs on exercise performance and skeletal muscle function in juvenile *mdx* mice.

Relative to baseline, *mdx* mice that had received mCDCs showed no differences in exercise performance or force-producing capacity of skeletal muscle at the 1-year study endpoint. The dramatic protection against functional deterioration in *mdx* skeletal muscle likely reflects not only the cytoprotective effects of mCDCs but also their regenerative benefits. Indeed, histology and RNA-seq showed that mCDCs target fibrosis and regenerative responses in *mdx* skeletal muscle, which are thought to underlie the preservation of muscle function. The commercial product of human CDCs, deramiocel, has shown safety and efficacy in clinical studies of patients with advanced DMD, most of whom were already non-ambulatory when therapy was initiated. The discovery that early and repeated administration of mCDCs significantly delays DMD progression offers a new treatment paradigm and motivates expanded use of the cells, giving reason to wonder whether the therapy might not be better initiated soon after diagnosis.

In contrast to mCDCs, repeated administration of hCDC-EVs resulted in initial improvements, but diminishing therapeutic benefits over time. hCDC-EVs have been traditionally viewed as non-immunogenic, as evidenced by their efficacy in several preclinical animal models (Dawkins et al., 2022; De Couto et al., 2017). Despite this, we show that hCDC-EVs elicit an IgG response in *mdx* mice following six monthly doses. It is currently unclear what specific antigen triggers the xenogeneic immune response, and its identification is beyond the scope of this report. Administration of human EVs was performed intentionally for translational relevance. Contrary to hCDC-EVs, infusion of human CDCs to immune-competent mice would not have been therapeutically beneficial, as xenogeneic cells are rapidly cleared by the immune system (Harding et al., 2024). Consequently, human EVs are far less immunogenic than their parent cells. Although our findings challenge the idea that human EVs are non-immunogenic, this notion is unlikely to become a barrier to clinical translation as alloreactivity is not necessarily expected in humans (Prunevieille et al., 2021).

## Methods

### Animals and injections

Eight-week-old female *mdx* mice (C57BL/10ScSn-DMD^mdx^/J) were used in this study. Mice were housed under pathogen-free conditions in a temperature-controlled room with a 12 h photoperiod. A graded exercise test was performed on a rodent treadmill at baseline and bi-monthly intervals. mCDCs (2.5 × 10^5^ cells) or hCDC-EVs (2.0 × 10^9^ particles) were suspended in 100 μL of Iscove’s Modified Dulbecco’s Medium (IMDM) and injected into the femoral vein of *mdx* mice once monthly during the 12-month study period. The doses of mCDCs and hCDC-EVs were determined previously (Rogers et al., 2019). Vehicle control *mdx* mice received an equal volume of IMDM injected into the femoral vein once monthly. Serum was collected at baseline and bi-monthly from the submandibular vein. Cohorts of mice were euthanized at the 6- or 12-month time point, and tissues were collected for muscle physiology, histology, or frozen in liquid nitrogen and stored at −80°C for further analyses. A separate cohort of 8-week-old *mdx* mice was used to record baseline skeletal muscle function.

### Generation of CDCs and purification of CDC-EVs

A heart was procured from an 8-week-old female C57BL/10ScSn/J mouse, as described (Rogers et al., 2019). Briefly, the ventricles were cut into fragments <1 mm^3^, washed, and digested with 0.05% trypsin (Gibco). Individual fragments were seeded onto fibronectin-coated culture dishes (Corning) and cultured in growth media (IMDM) supplemented with 20% fetal bovine serum (Atlas Biologicals), 1% penicillin/streptomycin (Gibco), and 0.1% 2-mercaptoethanol (Gibco). Cells emerging from the explants were harvested using mild enzymatic digestion (TrypLE, Gibco) and cultured in ultra-low adherent flasks (Corning) for 3 days to form cardiospheres. Cardiospheres were then plated on fibronectin-coated culture dishes (Corning) to form a monolayer of cells (i.e., mCDCs) and expanded to passage 5 for animal administration. To produce hCDC-EVs, hCDCs were developed and cultured for research purposes, as described (Smith et al., 2007), under an approved Cedars-Sinai Institutional Review Board protocol (#00014713). hCDCs were expanded to passage 5, washed, and cultured in serum-free IMDM at physiologically low oxygen (i.e., 2% O_2_) for 24 h. Conditioned media was then collected, filtered using a 0.45 μm sterile filter, and hCDC-EVs were purified by ultrafiltration via centrifugation using a 100 kDa MWCO centrifugal filter (EMD Millipore). hCDC-EV concentration (particles per mL) was measured by dynamic light scattering and nanoparticle tracking analysis (NanoSight NS300) and stored in ready-to-use aliquots at −80°C.

### Graded exercise testing

Mice were placed inside an Exer-3/6 rodent treadmill (Columbus Instruments) equipped with a shock grid and elevated 5°, as described (Rogers et al., 2019). Briefly, during the acclimatization period, mice were left undisturbed for 30 min prior to engagement of the belt. Once the belt was engaged, mice were encouraged to familiarize themselves with walking on the treadmill belt, at 10 m/min, for an additional 20 min. Following acclimatization, the graded exercise protocol engaged, and the shock grid was activated (0.15 mA at 1 shock/sec). The graded exercise test used here is intended to induce volitional exhaustion by increasing the belt speed by 1 m/min^2^. Mice that rest on the shock grid for >10 s with gentle nudging were considered to have reached their maximal exercise capacity, and the graded exercise test was terminated. Total accumulated distance was recorded for each mouse.

### *In vitro* isolated skeletal muscle physiology

Whole skeletal muscle physiology was performed, as previously described (Rogers et al., 2019), with slight modification. Briefly, mice were deeply anesthetized by isoflurane inhalation, and a lateral midline incision was made into the skin of the left hindlimb. The soleus muscle was freely dissected, and its tendons of origin and insertion were ligated with a 3-0 silk suture, and then vertically mounted in a tissue bath containing a mammalian Ringer’s buffer of the following composition: (in mM) 137 NaCl, 5 KCl, 2 CaCl_2_, 1 MgSO_4_, 1 NaH_2_PO_4_, 24 NaHCO_3_, and 11 glucose. The buffer was constantly aerated with 95% O_2_ and 5% CO_2_ with pH and temperature maintained at 7.35 and 24°C, respectively. One end of the soleus muscle was secured to a clamp at the bottom of the tissue bath, and the other end was attached to a calibrated force transducer (Model 300C, Aurora Scientific). A micromanipulator connected to the system was used to adjust muscle length. Platinum wire electrodes were placed on each side of the muscle for direct muscle stimulation (Aurora Scientific), using 0.2 ms monophasic rectangular pulses of constant current delivered at supramaximal intensity. Muscle preload was incrementally adjusted until the optimal muscle length for maximal isometric twitch force (Lo) was reached. Lo was measured at 0.1 mm accuracy using a digital caliper. Isometric contractile properties were then determined at Lo. Peak twitch force was determined from a single pulse. Force-frequency relationships were measured at stimulus frequencies ranging from 5 to 180 pulses/s. The stimuli were presented in trains of 1 s duration with an interval of at least 1 min between each stimulus train. Muscle forces generated, including twitch and maximal tetanic force, were normalized to the estimated physiological cross-sectional area (CSA) of the muscle segment, using the following formula:

$$CSA=\frac{muscle\;weight}{1.056\times Lo}$$

The constant 1.056 represents the density of the muscle (in g/cm^3^), and Lo was corrected for muscle fiber length of the mouse soleus (0.71 of Lo) to estimate muscle-specific force (expressed as Newtons [N]/cm^2^).

### RNA purification and sequencing

Total RNA, including small RNAs of approximately 18 nucleotides and greater, was purified from soleus muscles using the miRNeasy Mini Kit (QIAGEN) according to the manufacturer’s recommended protocol. RNA concentration and quality were measured using a Qubit Fluorometer (Thermo Fisher Scientific) and 2100 Bioanalyzer (Agilent Technologies), respectively (Sanchez et al., 2022). Only samples with an RNA integrity number of 8 or higher were used for sequencing. A sequencing library was constructed using the xGen Broad-Range RNA Library Prep Kit (Integrated DNA Technologies). Library concentration and size were measured using a Qubit Fluorometer (Thermo Fisher Scientific) and 4200 TapeStation (Agilent Technologies), respectively. Libraries were then multiplexed and sequenced using a NovaSeq 6000 (Illumina) with 75 base pair single-end sequencing. Approximately 30 million reads were generated from each sample. Raw reads were mapped to the latest UCSC transcript set using Bowtie 2, and gene expression levels were estimated using RSEM. Trimmed mean of M-values was used to normalize gene expression. Differentially expressed genes were identified using edgeR software. Genes with greater than 1.5-fold change and *p* < 0.05 were considered differentially expressed. Pathway and network analyses were performed using Ingenuity Pathway Analysis (IPA) software. The canonical pathways generated by IPA are the most significant for the datasets. Fischer’s exact test with a false discovery rate option was used to calculate the significance of each pathway. Raw sequencing data were deposited in Gene Expression Omnibus: GSE289126.

### Histology

The soleus from each mouse was processed, as previously described (Rogers and Otis, 2017). Briefly, tissues were embedded in OCT compound and frozen in 2-methylbutane pre-cooled in liquid nitrogen, and then stored at −80°C until cryosectioning. Serial sections were cut at the mid-belly in the transverse plane (Hong et al., 2024). All sections were cut between 5 and 8 μm using a cryostat (CM3050S, Leica) and adhered to Superfrost Plus microscope slides (Fisher Scientific). Cryosections were fixed with 10% neutral buffered formalin for 10 min prior to Picrosirius red staining (Sigma-Aldrich). Histological slides were imaged using an Aperio AT Turbo slide scanner (Leica) at 20× magnification. Quantification of fibrosis was determined by the area of red staining relative to yellow staining of the entire tissue section using QuPath v.0.4.3 software.

### Indirect enzyme-linked immunosorbent assay

Measurement of mouse anti-hCDC-EV IgG was performed using an uncoated mouse IgG enzyme-linked immunosorbent assay kit (Thermo Fisher Scientific, #88-50400-22) with slight modification. Briefly, the coating solution was prepared containing a coating buffer (provided by the kit) and hCDC-EVs (in lieu of the capture antibody), according to the manufacturer’s recommended protocol, and added to a clear 96-well plate. Following an overnight incubation at 4°C, the coating solution was aspirated, and the wells were washed once with a prepared wash buffer. A blocking buffer was then added to each well for 1 h at room temperature, aspirated, and samples (e.g., mouse sera or anti-CD63 antibody) were incubated in each well for 2 h at room temperature. Following sample incubation, samples were aspirated, and the wells were washed 5 times with a prepared wash buffer. A detection antibody solution (containing an HRP-conjugated anti-mouse IgG) was then added to each well for 2 h at room temperature, aspirated, and the wells were washed 5 times with a prepared wash buffer. A TMB substrate solution was then added to each well for 30 min at room temperature, and then an equal volume of stop solution was added. Absorbance was measured at 450 nm using a SpectraMax iD3 (Molecular Devices) microplate reader.

### *Ex vivo* optical imaging

hCDC-EVs were fluorescently labeled using BODIPY Ceramide (Invitrogen), according to the manufacturer’s recommended protocol. Excess dye was removed by filtration using Exosome Spin Columns (MW 3000, Invitrogen), according to the manufacture’s recommended protocol. Fluorescently labeled or unlabeled hCDC-EVs were injected into the femoral vein of *mdx* mice. Tissues were collected 24 h later, washed with ice-cold phosphate-buffered saline, and imaged using a Xenogen IVIS Spectrum Imaging System (Rogers et al., 2021).

### Statistical analyses

Data were collected by investigators blinded to group assignments and are represented as mean ± standard error of the mean. Physiological assessments (e.g., exercise testing and muscle function) were performed on consecutive days at each respective study endpoint. Sample sizes were 6–10 mice per condition or time point. Histological assessment was performed at once on all specimens. Sample sizes were 5–6 per condition or time point. Enzyme-linked immunosorbent assay was performed once with 3 biological replicates per condition. Statistical differences were determined by an independent t test (one-tailed), one-way ANOVA, or repeated measures ANOVA using Prism v.9 (GraphPad) software. In the case of datasets with non-normal distribution and unequal variance, a permutation test was performed using R Studio. Statistical significance was accepted at *p* ≤ 0.05.

### Study approval

All animal procedures were approved by the Institutional Animal Care and Use Committee at Cedars-Sinai Medical Center.

## Acknowledgments

We gratefully acknowledge expert Illumina sequencing by the Applied Genomics, Computational, and Translational Core at Cedars-Sinai Medical Center and bioinformatics by Xiangming Ding at TACGenomics. Financial support for this research project was provided by the 10.13039/100000002National Institutes of Health (R01 HL155346 to E.M.) and the 10.13039/100005202Muscular Dystrophy Association (DG578294 to R.G.R.). General laboratory support was provided by the 10.13039/100000002National Institutes of Health (R01 HL167921 to R.G.R.). E.M. holds the Mark S. Siegel Family Foundation Distinguished Chair of the Cedars-Sinai Medical Center.

## Author contributions

Conceptualization, R.G.R. and E.M.; experimental design, R.G.R. and E.M.; data collection, R.G.R., J.A., M.F., A.O., L.S., J.A., J.Z., N.M., A.C., and J.V.; data analysis, R.G.R.; manuscript drafting, R.G.R.; manuscript editing, R.G.R. and E.M.; funding, R.G.R. and E.M.; final approval, R.G.R.

## Declaration of interests

E.M. owns founder’s equity in Capricor Therapeutics.
